# Eating Habits of Children Born after Maternal Bariatric Surgery

**DOI:** 10.3390/nu12092577

**Published:** 2020-08-25

**Authors:** Karolien Van De Maele, Charlotte De Geyter, Yvan Vandenplas, Inge Gies, Roland Devlieger

**Affiliations:** 1 Pediatric Endocrinology, KidZ Health Castle, UZ Brussel, Vrije Universiteit Brussel, 1090 Brussels, Belgium; karolien.vandemaele@uzbrussel.be (K.V.D.M.); inge.gies@uzbrussel.be (I.G.); 2 Research Unit Organ Systems, Department of Development and Regeneration, Catholic University of Leuven, 3000 Leuven, Belgium; 3 Research Unit GRON, Vrije Universiteit Brussel, 1090 Brussels, Belgium; Yvan.Vandenplas@uzbrussel.be; 4 KidZ Health Castle, UZ Brussel, Vrije Universiteit Brussel, 1090 Brussels, Belgium; charlotte.degeyter@uzbrussel.be; 5 Department of Obstetrics and Gynecology, University Hospital of Leuven, 3000 Leuven, Belgium

**Keywords:** maternal obesity, maternal bariatric surgery, childhood diet

## Abstract

Mothers who underwent bariatric surgery (BS) before pregnancy have worrisome eating habits, but little is known about the eating habits of their offspring. EFFECTOR is a cross-sectional, long-term follow-up study of 4–11-year-old children born from mothers that underwent bariatric surgery before pregnancy (*n* = 36), mothers with overweight/obesity (OW/OB) in a control group (*n* = 71), and mothers with a normal weight (NW) in a second control group (*n* = 35). Data on anthropometry and on eating habits obtained through a Food Frequency Questionnaire were collected prospectively. The children’s body mass index (BMI) scores significantly correlated with maternal pre-pregnancy BMI. The prevalence of overweight and obesity was the highest in children of the BS group (38.9% vs. 15.5% for children of the OW/OB group and 5.7% for those of the NW group; *p* = 0.004). Meal-skipping behavior was comparable between the groups. There was no difference in fruit and vegetable consumption. The BS group consumed more low-calorie sweetened beverages compared to the NW group (*p* = 0.01) but less fruit juice compared to the NW and OW/OB groups (*p* = 0.01). Our results may indicate a sugar-avoiding behavior in children of the BS group, fitting dietary maternal habits in a strategy to prevent dumping syndrome. In conclusion, maternal pre-pregnancy bariatric surgery does not alter unhealthy eating behaviors and the risk of development of overweight during childhood in their children.

## 1. Introduction

Parental obesity is a known risk factor for the development of childhood obesity and contributes to a complex interaction between different biological, environmental, socio-economical, and behavior factors [1,2,3]. On the one hand, the family food environment plays a role in the development of certain food preferences and eating habits that might lead to obesity in children [1,2]. On the other hand, there are multiple other contributing factors such as (epi)genetic alterations and income and education level of the parents [3].

Obesity during certain critical periods, such as pregnancy, has been suggested to be more influential than in other periods of life [3]. Maternal obesity during pregnancy results in an increased risk for obesity in her children [4,5,6,7,8,9,10]. Since lifestyle interventions are often insufficient to obtain sustainable weight loss, bariatric surgery has gained more popularity in women at childbearing age [11,12].

However, data on the eating pattern of women after bariatric surgery remain scarce. Some studies report that unhealthy diet and irregular meal patterns persist after surgery, despite a limited food intake [13,14]. Opposite findings are reported by other authors, describing improved healthy eating patterns and child feeding practices in women who underwent bariatric surgery [15].

According to the WHO, 18% of children aged 5 to 19 years have overweight or obesity [16]. Therefore, it is crucial to detect and address childhood obesity at an early stage, certainly in particular risk groups such as parents with obesity and maternal bariatric surgery, since the problem is likely to persist into adulthood [17], leading to a vicious circle of obesity.

Little is known regarding the eating pattern and dietary choices of the offspring of mothers who underwent bariatric surgery before pregnancy. Additional information on this topic could provide insight in the ideal starting point for an intervention in the offspring born after bariatric surgery. Therefore, we prospectively studied the dietary pattern of the offspring of mothers who underwent bariatric surgery before pregnancy and compared them to those of the children of both a group of women who had overweight or obesity (OW/OB) and a group of women who had a normal weight (NW) during pregnancy.

## 2. Materials and Methods

The EFFECTOR study includes 143 children and is designed as a cross-sectional cohort follow-up study, investigating the offspring of different maternal cohorts [18]. The study was registered at ClinicalTrials.gov (NCT02992106) after obtaining approval by the Ethics Committee of the UZ Brussels and the UZ Leuven/KU Leuven. A written informed consent was obtained from all parents, and each child received an age-appropriate assent.

Thirty-six children born after maternal bariatric surgery (BS group) were compared to two control groups: (i) the offspring of mothers who had overweight/obesity (body mass index (BMI) ≥ 25 kg/m^2^) at the start of pregnancy (*n* = 71) (OW/OB group) and (ii) the offspring of mothers who had a normal BMI (BMI ≥ 18.5 and ≤ 25 kg/m^2^) at the start of pregnancy (*n* = 36) (NW group). The mothers were aged between 19 and 41 years at the moment of their pregnancy (mean 29.5 years). One child was excluded from the last group because the parents did not complete the questionnaires.

### 2.1. Outcomes

According to the study protocol [18], data were collected prospectively during a single home visit. All visits were performed by the same trained pediatrician (KVDM).

Standing height was measured with a stadiometer (seca 217), weight and body fat percentage were assessed by bio-electrical impedance analysis (BIA; Tanita MC-780U). BMI was calculated and expressed as SD score according to national reference data [19]. Skin fold measurements at four sites were performed with a Harpenden skinfold caliper (Baty international, RH15 9LB England) according to the international society for the advancement of kinanthropometry [20]. The girth of the waist circumference was measured with a flexible Lufkin steel anthropometric tape (Lufkin W606PM). Each anthropometric measurement was taken twice, and the mean was calculated. If the difference between the first and the second measure was >5% for skinfolds and >1% for the circumference, the measurement was repeated a third time, and the mean of the two nearest values was used. Subcutaneous fat thickness was measured at the sites of the four skinfolds with a portable ultrasound device with a linear probe 7.5 Mhz (Mindray, Diagnostic Ultrasound System, model M 7).

Additional data were collected through validated parental questionnaires. A questionnaire on socio-demographic characteristics was specifically designed for the study. Furthermore, we used a 47-item Food Frequency Questionnaire (FFQ) which was validated for the studied population [21,22,23].

### 2.2. Statistical Analysis

All statistical analyses were performed using SPSS version 25 (IBM Corp. Released 2017. IBM SPSS Statistics for Windows, Version 25.0. Armonk, NY, USA: IBM Corp.). Data are presented as mean ± standard deviation (SD). Descriptive statistics were used to describe the population characteristics. For continuous variables, after testing for normality, one-way ANOVA tests were done. Correlation was tested using Pearson Correlation tests. Pearson Chi Square tests were used for the comparison of categorical variables when the assumptions were met. Whenever the assumptions for the Pearson Chi Square were not met (cell counts less than 5), Fisher Exact tests were performed. If ANOVA or Chi Square testing was done over the three groups and if a difference was found, a post-hoc testing was performed to highlight between which groups the difference existed. Post-hoc tests were done using a Bonferroni correction to avoid type-1 error in case of multiple testing; *p* < 0.05 was considered statistically significant.

## 3. Results

### 3.1. Study Population

Data from 142 children were included in the analyses, and their baseline characteristics are represented in Table 1. The children of the BS group were younger but had higher weight SD scores and BMI SD scores than the children of the normal-weight control group (*p*-values for age, weight SD scores, and BMI SD scores were, respectively, <0.001, 0.005, and 0.007). Parental education levels were also lower in the BS group compared to the normal-weight control group (*p <* 0.001). Children of the BS group had the lowest absolute values of height (*p <* 0.001), weight (*p* < 0.001), and neck (*p <* 0.001), waist (*p* = 0.02), and hip circumferences (*p <* 0.001) in comparison to the groups with normal weight and with overweight/obesity; these differences can be explained by the age difference between the groups.

**Figure 1:**
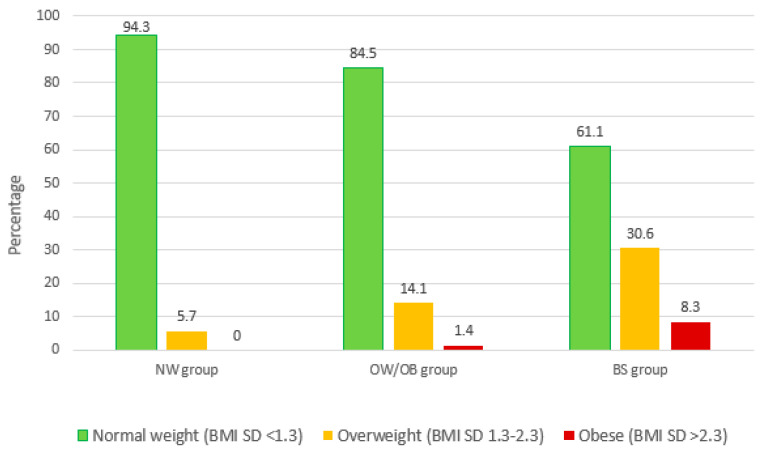
Prevalence of weight categories for the children, based on the maternal groups.

In the study population of 142 children, there was a prevalence of 16.2% of children with overweight (BMI SD score of 1.3–2.3) and of 2.8% of children with obesity (BMI SD score >2.3). The prevalence differs based on the study subgroups. In the BS group, 22/36 (61.1%) of the children had a normal weight, compared to 33/35 (94.3%) of the children in the NW group and 60/71 (84.5%) of the children in the OB/OW group (overall *p* = 0.004; post-hoc analysis resulted in a difference only between BS and NW) (Figure 1).

The BMI SD scores in our study population significantly correlated with all other anthropometrical parameters (percentage total body fat, neck, waist, and hip circumferences, ultrasound measures of subcutaneous fat mass, and sum of skinfolds; *p <* 0.001). The BMI SD scores were therefore used as the main outcome in the correlation analysis. The BMI SD scores correlated significantly with maternal pre-pregnancy BMI (R = 0.364, *p <* 0.001) and with actual paternal BMI (R 0.297, *p* = 0.001), but not with actual maternal BMI (R = 0.151, *p* = 0.075).

Bariatric surgery procedures performed in these women included: Laparoscopic Roux-en-Y Gastric Bypass (LRYGB) (*n* = 24; 66.7%), Laparoscopic Adjustable Gastric Banding (LABG) (*n* = 10; 27.8%), or Scopinaro Procedure (*n* = 2; 5.6%). The mean interval between surgery and pregnancy was almost 4 years (mean 47.4 months; range 2–113 months); 8/36 (22.2%) women became pregnant within the first year after BS. The mean BMI before surgery was 43.0 kg/m^2^ and declined to 29.5 kg/m^2^ before pregnancy. The pre-pregnancy BMI of the mothers in the BS group was higher than the BMI of the mothers in the NW group but lower than that of the mothers in the OW/OB group (29.5 for BS mothers versus 21.8 for NW and 32.4 for OW/OB mothers; overall *p* < 0.001).

In Table 2, we listed the average daily intake of the main food groups for our study groups and the median intake as estimated by the Belgian National Food Consumption Survey.

### 3.2. Meal Pattern

In our study groups, 13/141 (9.2%) of the children did not regularly consume breakfast (≤4 times a week); 5/13 (38.5%) breakfast skippers were female (versus 61.5% of males; *p* = 0.37). Children who had breakfast ≤4 times a week had a higher BMI SD score than children eating breakfast on a regular basis (*n* = 128; mean BMI SDS respectively 0.77 vs. −0.03; *p* = 0.023). No significant differences were found for the other anthropometric parameters, nor a significant correlation between breakfast skipping and physical activity, duration of sleep, or screen time.

Meal skipping observed in children based on maternal subgroups is shown in Table 3. No significant differences between the groups were observed.

### 3.3. Food Intake Organized by Food Groups

#### 3.3.1. Meat and Fish

There were no significant differences in frequency or amount of meat (anything but poultry) or fish consumption. Children in the BS group did consume poultry less frequently compared to those in the OW/OB group (25% more than 75 g vs. 56.7% for OW/OB and 47.1% for NW groups; overall *p* = 0.008) (Table 4).

#### 3.3.2. Dairy Products

No significant differences were found in the amount or frequency of milk consumption. Children in the NW group consumed sugared yoghurt more frequently in comparison to BS children (95% > 65 g vs. 53.3% for BS children and 81.8% for OW/OB children; overall *p* = 0.01, Table 4).

#### 3.3.3. Vegetables and Fruits

No significant differences across groups were observed in the consumption of vegetables and fruit (Table 4). We display the numbers for prepared vegetables and fresh fruit since the reported amounts of consumed raw vegetables and canned fruits were very low.

#### 3.3.4. Beverages

Consumption of fruit juices was lower for the children in the BS group than for those in both control groups; consumption for both control groups was similar (8.3% vs. 37.1% for NW and 30.0% for OW/OB children; overall *p* = 0.01) (Table 5). The children in the BS group consumed more artificial, low-calorie sweetened beverages in comparison to those in the NW control group (mostly zero and light soft drinks) (38.9% vs. 26.8% of OW/OB and vs. 8.6% of NW children; overall *p* = 0.01), and 19.4% of them did that daily (vs. 13% of OW/OB children; no consumption for NW children). Moreover, 18% of BS children consumed more than 600 mL of these beverages per day (Table 5).

No significant differences were found in the frequencies and quantities of water and sugar-sweetened beverages consumption across the groups (Table 5).

#### 3.3.5. Snacks

In all groups, children consumed snacks quite often; however, few differences between groups were found regarding the frequency of chocolate and sweet or salty snacks eating (Table 6). Children in the BS group did consume the smallest quantity of salty snacks compared to those in the OW/OB control group (55.9% >25 g vs. 82.5% for OW/OB and 82.4% for NW children; overall *p* = 0.01) (Table 6).

Children in the NW group consumed more frequently chocolate mousse and ice cream compared to children in the OW/OB group (28.6% at least two times a week vs. 7.2% of OW/OB children and 13.9% of BS children; overall *p* = 0.01). Children in the BS group consumed significantly more milk desserts, such as (rice) pudding, flan, or milkshakes compared to those in the OW/OB control group but not to those in the NW control group (25% at least two times a week vs. 5.8% of OW/OB children and 17.6% of NW children; overall *p* = 0.01) (Table 6).

## 4. Discussion

To the best of our knowledge, this is the first report on the dietary habits of children born after maternal pre-pregnancy bariatric surgery. We found differences in the eating behavior of these children when comparing them to children born from mothers with high or normal pre-pregnancy BMI. Parental eating habits are known to influence the eating habits of their children [1]. An increased risk of food overconsumption for children has been described, especially when there is a combination of dietary restrictions and parental disinhibited eating patterns [18,19]. In comparison to a group of children from mothers with a normal weight before pregnancy, children of the BS group consumed more low-calorie sweetened beverages and less sugared yoghurts. Compared to the offspring of women with overweight or obesity before pregnancy, children of the BS group consumed smaller amounts of salty snacks and poultry but more milk desserts. The children in the BS group drank juice less frequently compared to both control groups.

Although these differences in dietary intake may appear futile, they could be related to the differences in BMI and body composition between the children in the different study groups. Indeed, 39% of the children in the BS group have overweight or obesity at the age of 6.5 years, compared to 15% and 6% of children in the OW/OB and NW groups, respectively.

For the 142 children studied, BMI SD scores significantly correlated with maternal pre-pregnancy BMI and with the paternal BMI at follow-up. A strong association between maternal obesity before pregnancy and childhood obesity is reported to be due to a combination of different in utero programming mechanisms [4,6,25]. Paternal obesity might play an additional role through epigenetic alterations, but this needs further confirmation [26]. Since there is also a correlation between current parental obesity and childhood obesity, the actual food and family environment interfere as well [1,3].

### 4.1. Meal Pattern

One of our concerns was meal-skipping behavior in the children of the BS group. Several studies have highlighted the importance of a regular meal pattern and, more particularly, the key role of breakfast. A regular breakfast consumption protects against the development of adiposity [27,28,29,30,31,32]. Skipping breakfast is a well-known risk factor for overweight and obesity in school-age children [33,34]. Our results show that 90.8% of the 142 children had breakfast at least five times a week, which is in line with national and international data [24,35]. Children skipping breakfast have higher BMI SD scores than children having regular breakfast. Significant higher waist circumference and central adiposity are reported in children skipping breakfast [36,37]. There is no consistency in the literature regarding sex-based differences in breakfast-skipping behavior: some studies show a dominance of girls with this habit [38,39,40], whereas others report it more frequently in boys [41], and some authors find it more often in both girls and boys with obesity [42,43,44]. We did not find any sex differences.

We hypothesized that children of the BS group would be at risk for meal-skipping behavior, because meal skipping and increased snacking behavior are described in women after maternal bariatric surgery, and parental eating habits influence the eating habits of their children [1,13,14]. Our data do not support this hypothesis, since there were no statistically significant differences across the groups regarding meal skipping. A small number of BS children did skip meals (19.4% skipped breakfast, 8.3% skipped lunch or dinner). However, the small number of children in each group may bias the estimation of meal-skipping behavior. The younger age of the children in the BS group compared to both control groups (6.5 years in the BS group vs. 10.2 years in the OW/OB group and 10.6 years in the NW group) may have introduced a bias as well, since meal-skipping behavior increases with age [24,38,39]. Lunch-skipping behavior has mostly been studied in adolescents, thus in children older than those in our study groups [45,46,47]. The scarce data on lunch skipping in younger children indicate mostly concerns on micronutrient intake and do not mention a correlation with body fat mass [48,49].

### 4.2. Food Intake

No differences in meat, except for poultry, and fish consumption across the study groups were observed. The children in the BS group had a lower poultry intake compared to those in the OW/OB group but not compared to those in the NW group. Poultry consumption correlates with a healthy and balanced diet and is associated with a decreased risk for the development of overweight and obesity when part of a balanced diet [50,51]. Milk consumption was comparable across all groups, but sugared yoghurt was consumed less by the children in the BS group. Data on the correlation between dairy products consumption and weight status of children are conflicting: some authors suggest a beneficial effect of dairy food consumption on body weight, but it is also known that a high protein intake in early life correlates with childhood obesity [52,53,54,55].

Fruit and vegetable consumption reduces the risk of childhood obesity [56]. According to our findings, fruit and vegetable consumption was low in the three groups and without any difference between them. BS children had a lower frequency of juice consumption compared to children in both control groups. Results from previous studies are inconsistent when it comes to fruit juice consumption. Some studies report a positive association with adiposity, although only in young children under the age of five; however, when controlled for total energy intake, fruit juice consumption cannot be associated with central adiposity [57,58,59,60,61,62,63,64,65].

Increased intake of sugar, mainly sugar-sweetened beverages, is thought to play a key role in the rise of obesity [66,67,68,69,70,71]. However, we did not find significant differences in the frequencies of sugar-sweetened beverages consumption across groups, nor did we find correlations with anthropometric measurements. This is in contrast to several studies reporting a significant positive association between central adiposity and sugar-containing drinks [72,73,74,75,76,77,78]. However, 38.9% of the children in the BS group drank low-calorie sweetened beverages more than twice a week, in comparison to only 8.6% of children in the NW group. Contrary to what “light” suggests, these artificially sweetened beverages are suggested to stimulate weight gain [79,80,81]. The explanatory underlying hypothesis is compensatory overeating due to an inconsistent pairing between a sweet taste and caloric content [79]. Some studies report an adverse adipocyte programming after early life exposure to these low-calorie sweetened beverages, but the evidence is limited and conflicting [82,83,84].

Sweet snacks were frequently consumed by all groups. Daily consumption of biscuits and other sweet snacks in the Belgian population is reported to be 45% for 6–9-year-old children and 25% for 10–13-year-olds [24]. Children of the BS group consumed smaller amounts of salty snacks compared to children of the OW/OB control group, whose portion sizes were more comparable to those of the NW control group. There is no consistency in the literature about the impact of snacking and its relation with overweight and obesity, although some authors suggest that frequent meals, including snacking, have a beneficial impact on BMI [85,86,87,88]. The national food survey also noticed that people with a healthy BMI more often eat sweet snacks than people with abnormal BMI [24]. It has been shown that subjects with obesity are prone to underreport food consumption more than subjects with a healthy BMI [89]. Unhealthy foods are more frequently underreported than healthy foods [90,91,92,93].

The food intake pattern in the BS group, for example the combination of lower consumption of fruit juice and sugared yoghurt and higher consumption of low-calorie sweetened drinks, may indicate a sugar-avoiding behavior. This may fit in a maternal strategy to prevent dumping syndrome after surgery [94].

The originality of our study is its pioneering nature, since there are no other long-term data available on the dietary habits of the offspring of mothers who underwent bariatric surgery before pregnancy. The fact that we used validated questionnaires is an additional strength [21,22,23].

The major limitation of this study is the lack of information on the food intake of the parents at the moment of the study visit. Another limitation is the fact that the children in the BS group were significantly younger than the children in the two other groups (age 6.5 years vs. 10.2 and 10.6; *p <* 0.001). Because of the single study visit and cross-sectional design with different ages in the different groups, we might also have missed possible confounders or age influences.

In summary, children born after pre-pregnancy maternal BS showed a persistent risk for the development of overweight and obesity at childhood age and presented associated unhealthy eating behaviors. More research is needed to study the dietary habits of women after bariatric surgery and the correlation with the dietary habits of their offspring.

## Figures and Tables

**Table 1:** Overall population characteristics.

	NW (*n* = 35)	OW/OB (*n* = 71)	BS (*n* = 36)	*p*-Value	Post-Hoc
Age (years)	10.6 (0.2)	10.2 (1.9)	6.5 (1.3)	<0.001	BS < NW and OW/OB
Sex (female)	18 (51.4%)	37 (52.1%)	17 (47.2%)	0.89	-
Height (cm)	146.4 (5.4)	143.4 (13.0)	123.2 (9.6)	<0.001	BS < NW and OW/OB
Height (SD score)	0.42 (0.81)	0.36 (0.91)	0.64 (0.92)	0.31	-
Weight (kg)	35.6 (6.2)	37.6 (11.6)	26.3 (7.5)	<0.001	BS < NW and OW/OB
Weight (SD score)	−0.086 (0.84)	0.24 (0.95)	0.70 (1.27)	0.005	NW < BS
BMI (kg/m²)	16.5 (2.3)	17.9 (3.6)	17.1 (2.9)	0.09	-
BMI (SD score)	−0.42 (1.06)	0.68 (1.03)	0.47 (1.50)	0.007	NW < BS
Total body fat (%) (BIA)	20.2 (4.4)	22.5 (6.5)	23.4 (5.2)	0.06	-
Neck circumference (cm)	28.3 (1.8)	28.9 (2.7)	26.9 (1.8)	<0.001	BS < NW and OW/OB
Waist (cm)	60.7 (7.2)	62.8 (9.5)	57.8 (8.2)	0.02	BS < OW/OB
Hip (cm)	73.3 (5.2)	76.1 (10.5)	67.5 (8.8)	<0.001	BS < NW and OW/OB
Skinfold biceps (mm)	9.7 (4.7)	11.1 (5.7)	11.5 (5.1)	0.32	-
Skinfold triceps (mm)	11.9 (4.2)	13.4 (5.8)	12.4 (4.2)	0.32	-
Skinfold Subscapularis (mm)	7.5 (4.0)	9.0 (6.8)	8.6 (4.1)	0.47	-
Skinfold Supraspinalis (mm)	8.7 (5.3)	10.5 (7.6)	9.9 (5.8)	0.44	-
Sum of skinfolds (SSF, mm)	37.9 (17.2)	44.0 (24.7)	42.5 (17.6)	0.39	-
Hours of night sleep	9.8 (0.8)	9.6 (1.1)	10.3 (1.3)	0.009	OW/OB < BS
Screen time/day (h)	2.2 (1.2)	2.7 (1.1)	2.4 (1.1)	0.10	-
Physical activity/week (h)	3.5 (2.1)	3.5 (2.7)	2.6 (1.8)	0.18	-
Pre-pregnancy BMI	21.8 (1.9)	32.4 (4.1)	29.5 (5.0)	<0.001	NW < BS < OW/OB
Actual maternal BMI	23.1 (3.1)	31.4 (5.5)	30.4 (6.3)	<0.001	NW < BS and OW/OB
Mother education level: college or university	30 (85.7%)	54 (77.1%)	16 (44.4%)	<0.001	BS < NW and OW/OB
Actual paternal BMI	24.7 (3.2)	26.6 (4.5)	28.5 (4.8)	0.003	NW < BS
Father education level: college or university	24 (70.6%)	28 (41.8%)	7 (20.6%)	<0.001	BS and OW/OB < NW

Legend: BIA: Bioelectrical impedance analysis; BMI: Body Mass Index; BS: Bariatric Surgery; SD Score: Z-score according to national reference data; NW: normal weight; OB: obesity; OW: overweight; SCF: Subcutaneous Fat; SSF: Sum of Skin Folds; US: ultrasound.

**Table 2:** Overview of average nutritional consumption for the Belgian population (children 6–9 years old) [24] and our study groups.

	National Population	NW (*n* = 35)	OW/OB (*n* = 71)	BS (*n* = 36)
Water (/day)	589 mL	54.3% > 600 mL	47.9% > 600 mL	31.4% >6 00 mL
Other sugar-free beverages (/day)	24 mL	28 mL (±73 mL)	62 mL (±123 mL)	110 mL (±185 mL)
Fruit juice (/day)	90 mL	135 mL (±109 mL)	113 mL (±102 mL)	62 mL (±57 mL)
Sugar-Sweetened Beverages	145 mL 52% min 2x/week o/w 20% daily	112 mL (±111 mL) 29% min 2x/week o/w 12% daily	147 mL (±141 mL) 35% min 2x/week o/w 14% daily	200 mL (±198 mL) 28% min 2x/week o/w 19% daily
Vegetables (/day)	96 g	114 g (±57 g)	115 g (±63 g)	119 g (±50 g)
Fruit (/day) Daily	120 g 62%	133 g (±64 g) 71%	137 g (±52 g) 72%	121 g (±55 g) 50%
Meat (/day)	112 g	NA	NA	NA
Fish (/week)	44% <1x	46% <1x	67% <1x	67% <1x
Rest group (per day)	356 g (632 kcal)	NA	NA	NA
Biscuits and cake (/day)	58 g 86% >2x/week o/w 45% daily	48 g (±21 g) 87% >2x/week o/w 37% daily	49 g (±23 g) 86% >2x/week o/w 48% daily	42 g (±23 g) 86% >2x/week o/w 28% daily
Sweets and chocolate (/day)	40 g	NA	NA	NA

Legend: Min: minimum; NA: Not Applicable: the data could not be subtracted from the questionnaire; o/w: of which.

**Table 3:** Meal pattern of the children.

	NW (*n* = 35)	OW/OB (*n* = 71)	BS (*n* = 36)	*p*-Value
Breakfast				0.83
Daily	31/35 (88.6%)	59/70 (84.3%)	29/36 (80.6%)	
5–6 times/week	2/35 (5.7%)	5/70 (7.1%)	2/36 (5.5%)	
≤4 times/week	2/35 (5.7%)	6/70 (8.6%)	5/36 (13.9%)	
Lunch				0.72
Daily	34/35 (97.1%)	67/70 (95.7%)	33/36 (91.7%)	
5–6 times/week	1/35 (2.9%)	3/70 (4.3%)	3/36 (8.3%)	
Dinner				0.05
Daily	35/35 (100%)	70/70 (100%)	33/36 (91.7%)	
5–6 times/week			1/36 (2.8%)	
≤4 times/week			2/36 (5.5%)	

**Table 4:** Consumption of the main food groups by the children.

	NW (*n* = 35)	OW/OB (*n* = 71)	BS (*n* = 36)	*p*-Value
**Meat**				
**Frequency per week**				0.53
≥5 times	5/35 (14.3%)	13/70 (18.6%)	9/36 (25 %)	
≤4 times	30/35 (85.7%)	57/70 (81.4%)	27/36 (75%)	
**Quantity per average consumption day**				0.36
>75 g	18/32 (56.3%)	43/69 (62.3%)	16/34 (47.1%)	
≤75 g	14/32 (43.8%)	26/69 (37.7%)	18/34 (52.9%)	
**Poultry**				
**Frequency per week**				0.11
<2 times	17/35 (48.6%)	21/71 (29.6%)	16/36 (44.4%)	
≥2 times	18/35 (51.4%)	50 /71 (70.4%)	20/36 (55.6%)	
**Quantity per average consumption day**				0.008 Post-hoc: BS < OW/OB
>75 g	16/34 (47.1%)	38/67 (56.7%)	9/36 (25%)	
≤75 g	18/34 (52.9%)	29/67 (43.3%)	25/36 (75%)	
**Fish**				
**Frequency per week**				0.10
<1 time	16/35 (45.7%)	46/69 (66.7%)	24/36 (66.7%)	
≥1 time	19/35 (54.3%)	23/69 (33.3%)	12/36 (33.3%)	
**Quantity per average consumption day**				0.21
>75 g	13/29 (44.8%)	24/41 (58.5%)	10/27 (37%)	
≤75 g	16/29 (55.2%)	17/41 (41.5%)	17/27 (63%)	
**Milk**				
**Frequency per week**				0.81
<1 time	13/35 (37.1%)	28/70 (40.0%)	16/36 (44.4%)	
≥2 times	22/35 (62.9%)	42/70 (60.0%)	20/36 (55.6%)	
**Quantity per average consumption day**				0.38
>200 mL	15/24 (62.5%)	23/45 (51.1%)	10/24 (41.7%)	
≤200 mL	9/24 (37.5%)	22/45 (48.9%)	14/24 (58.3%)	
**Sugared yoghurt**				
**Frequency per week**				0.91
<1 time	29/35 (82.9%)	61/71 (85.9%)	31/36 (86.1%)	
≥2 times	6/35 (17.1%)	10/71 (14.1%)	5/36 (13.9%)	
**Quantity per average consumption day**				0.01 Post-hoc: BS < NW
>65 g	19/20 (95.0%)	27/33 (81.8%)	8/15 (53.3%)	
≤65 g	1/20 (5.0%)	6/33 (18.2%)	7/15 (46.7%)	
**Fruit**				
**Frequency per week**				0.97
≥5 times	27/35 (77.1%)	54/70 (77.1%)	27/36 (75%)	
≤4 times	8/35 (22.9%)	16/70 (22.9%)	9/36 (25%)	
**Quantity per average consumption day**				0.55
>75 g	31/35 (88.6%)	65/69 (94.2%)	33/36 (91.7%)	
≤75 g	4/35 (11.4%)	4/69 (5.8%)	3/36 (8.3%)	
**Vegetables**				
**Frequency per week**				0.38
≥5 times	19/35 (54.3%)	36/71 (50.7%)	14/36 (38.9%)	
≤4 times	16/35 (45.7%)	35/71 (49.3%)	22/36 (61.1%)	
**Quantity per average consumption day**				0.32
>180 g	5/35 (14.3%)	4/68 (5.9%)	2/33 (6.1%)	
≤180 g	30/35 (85.7%)	64/68 (94.1%)	31/33 (93.9%)	

Legend: g: grams; ml: milliliters.

**Table 5:** Beverage consumption.

	NW (*n* = 35)	OW/OB (*n* = 71)	BS (*n* = 36)	*p*-Value
**Fruit juice**				
**Frequency per week**				0.01 Post-hoc: BS < OW/OB and NW
≥2 days	13/35 (37.1%)	21/70 (30.0%)	3/36 (8.3%)	
≤1 day	22/35 (62.9%)	49/70 (70.0%)	33/36 (91.7%)	
**Quantity per average consumption day**				0.53
>200 mL	8/28 (28.6%)	19/49 (38.8%)	5/19 (26.3%)	
≤200 mL	20/28 (71.4%)	30/49 (61.2%)	14/19 (38.9%)	
**Low-calorie sweetened beverages**				
**Frequency per week**				0.01 Post-hoc: BS > NW
≥2 days	3/35 (8.6%)	19/71 (26.8%) o/w 9/71 (13%) daily	14/36 (38.9%) o/w 7/36 (19%) daily	
≤1 day	32/35 (91.4%)	52/71 (73.2%)	22/36 (61.1%)	
**Quantity per average consumption day**				0.94
>200 mL	7/13 (53.8%)	19/34 (55.9%)	8/17 (47.1%) o/w 3/17 (18%) > 600 mL	
≤200 mL	6/13 (46.2%)	15/34 (44.1%)	9/17 (52.9%)	
**Sugar-sweetened beverages**				
**Frequency per week**				0.73
≥2 days	10/34 (29.4%) o/w 4/34 (12%) daily	25/71 (35.2%) o/w 10/71 (14%) daily	10/36 (27.8%) o/w 7/36 (19%) daily	
≤1 day	24/34 (70.6%)	46/71 (64.8%)	26/36 (72.2%)	
**Quantity per average consumption day**				0.35
>200 mL	13/26 (50%)	23/50 (46%) o/w 4/50 (8%) > 400	6/20 (30.0%) o/w 2/20 (10%) > 400	
≤200 mL	13/26 (50%)	27/50 (54%)	14/20 (70%)	
**Water**				
**Frequency per week**				0.43
≤6 days	2/35 (5.7%)	3/71 (4.2%)	4/36 (11.1%)	
Daily	33/35 (94.3%)	68/71 (95.8%)	32/36 (88.9%)	
**Quantity per average consumption day**				0.14
<600 mL/day	16/35 (45.7%)	37/71 (52.1%)	24/35 (68.6%)	
>600 mL/day	19/35 (54.3%)	34/71 (47.9%)	11/35 (31.4%)	

**Table 6:** Snack consumption by the children.

	NW (*n* = 35)	OW/OB (*n* = 71)	BS (*n* = 36)	*p*-Value
**Chocolate**				
**Frequency per week**				0.86
≥2 times	9/35 (25.7%)	22/70 (31.4%)	10/36 (27.8%)	
≤1 time	26/35 (74.3%)	48/70 (68.6%)	26/36 (72.2%)	
**Quantity per average consumption day**				0.80
>25 g	10/31 (32.3%)	21/55 (38.2%)	10/31 (32.3%)	
≤25 g	21/31 (67.7%)	34/55 (61.8%)	21/31 (67.7%)	
**Sweet snacks**				
**Frequency per week**				1.0
≥2 times	31/35 (88.6%)	61/71 (85.9%)	31/36 (86.1%)	
≤1 time	4/35 (11.4%)	10/71 (14.1%)	5/36 (13.9%)	
**Quantity per average consumption day**				0.19
>25 g	26/35 (74.3%)	45/66 (68.2%)	19/35 (54.3%)	
≤25 g	9/35 (25.7%)	21/66 (31.8%)	16/35 (45.7%)	
**Salty snacks**				
**Frequency per week**				0.84
≥2 times	7/35 (20%)	18/71 (25.4%)	9/36 (25%)	
≤1 time	28/35 (80%)	53/71 (74.6%)	27/36 (75%)	
**Quantity per average consumption day**				0.01 Post-hoc: BS < OW/OB
>25 g	28/34 (82.4%)	52/63 (82.5%)	19/34 (55.9%)	
≤25 g	6/34 (17.6%)	11/63 (17.5%)	16/34 (44.1%)	
**Chocolate mousse/ice cream**				
**Frequency per week**				0.01 Post-hoc: NW > OW/OB
≥2 times	10/35 (28.6%)	5/69 (7.2%)	5/36 (13.9%)	
≤1 time	25/35 (71.4%)	64/69 (92.8%)	31/36 (86.1%)	
**Quantity per average consumption day**				0.34
>65 g	19/29 (65.5%)	29/49 (59.2%)	14/30 (46.7%)	
≤65 g	10/29 (34.5%)	20/49 (40.8)	16/30 (53.3%)	
**Milk desserts**				
**Frequency per week**				0.01 Post-hoc: BS > OW/OB
≥2 times	6/34 (17.6%)	4/69 (5.8%)	9/36 (25%)	
≤1 time	28/34 (82.4%)	65/69 (94.2%)	27/36 (75%)	
**Quantity per average consumption day**				0.59
>65 g	13/16 (81.2%)	34/41 (82.9%)	19/26 (73.1%)	
≤65 g	3/16 (18.8%)	7/41 (17.1%)	7/26 (26.9%)	
